# QuickStats

**Published:** 2013-12-20

**Authors:** Linda F. McCaig, Michael Albert

**Figure:**
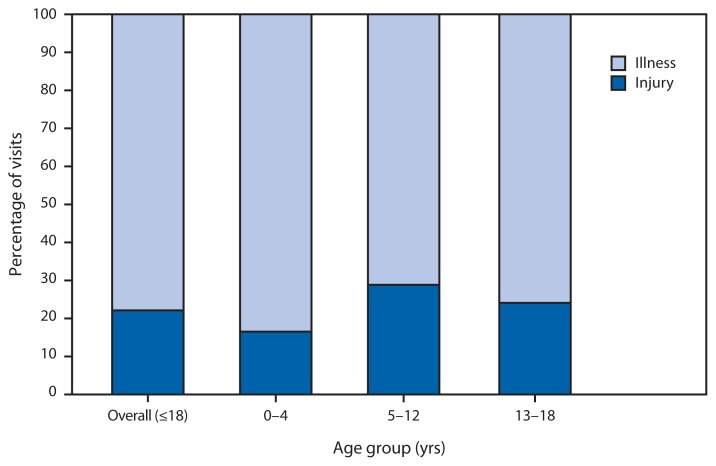
Percentage of Hospitalizations After Emergency Department Visits Resulting from Illness and Injury Among Persons Aged ≤18 Years, by Age Group — National Hospital Ambulatory Medical Care Survey, United States, 2007–2010^*^ ^*^ Percentages are 4-year annual averages.

During 2007–2010, emergency department visits by children resulting in hospital admission were more likely to be related to illness (78%) than injury (22%). This pattern applied to persons aged 0–4 years, 5–12 years, and 13–18 years, with the greatest difference observed among children aged 0–4 years, for whom 84% of visits resulting in hospital admission were related to illness, compared with only 16% related to injury.

**Source:** National Hospital Ambulatory Medical Care Survey, 2007–2010. Available at http://www.cdc.gov/nchs/ahcd.htm.

